# Lessons from an Evaluation of a Provincial-Level Smoking Control Policy in Shanghai, China

**DOI:** 10.1371/journal.pone.0074306

**Published:** 2013-09-10

**Authors:** Xiang Li, Junling Gao, Zhixing Zhang, Minqi Wei, Pinpin Zheng, Eric J. Nehl, Frank Y. Wong, Carla J. Berg

**Affiliations:** 1 Key Laboratory of Public Health Safety, Ministry of Education, School of Public Health, Fudan University, Shanghai, China; 2 Health Inspection Institute of Changning District, Shanghai, China; 3 Center of Diseases Control in Minhang District, Shanghai, China; 4 Department of Behavioral Sciences & Health Education, Emory University Rollins School of Public Health, Atlanta, Georgia, United States of America; The University of Auckland, New Zealand

## Abstract

**Background:**

The Shanghai Public Places Smoking Control Legislation was implemented in March 2010 as the first provincial-level legislation promoting smoke-free public places in China.

**Objective:**

To evaluate the compliance with this policy as well as its impact on exposure to secondhand smoke (SHS), respiratory symptoms, and related attitudes among employees in five kinds of workplaces (schools, kindergartens, hospitals, hotels, and shopping malls).

**Methods:**

A cross-sectional survey was conducted six months before and then six months after the policy was implemented. Five types of occupational employees from 52 work settings were surveyed anonymously using multistage stratified cluster sampling.

**Results:**

Six months after implementation, 82% of the participants agreed that “legislation is enforced most of the time”. The percentage of self-reported exposure to secondhand smoke declined from round up to 49% to 36%. High compliance rates were achieved in schools and kindergartens (above 90%), with less compliance in hotels and shopping malls (about 70%). Accordingly, prevalence of exposure to SHS was low in schools and kindergartens (less than 10%) and high in hotels and shopping malls (40% and above). The prevalence of respiratory and sensory symptoms (e.g., red or irritated eyes) among employees decreased from 83% to 67%.

**Conclusions:**

Initial positive effects were achieved after the implementation of Shanghai Smoking Control legislation including decreased exposure to SHS. However, compliance with the policies was a considerable problem in some settings. Further evaluation of such policy implementation should be conducted to inform strategies for increasing compliance in the future.

## Introduction

Research into the adverse health effects of exposure to secondhand smoke (SHS) has consistently identified it as a public health hazard and cause of disease [1]. By the end of 2010, 28 countries had 100% comprehensive smoke-free policies, covering 11% of the world’s population [2,3]. Many countries have witnessed the successful enforcement of smoke-free policies. In Scotland, 99.4% of all premises complied with smoking bans in the first 3 months [4]. Similarly, the United Kingdom achieved 98% compliance, and Ireland achieved 94% compliance within a year [5]. High compliance rates have also been achieved in developing countries such as India, Mexico, and Uruguay [1,6,7]. Concurrently, successful implementation also leads to declines in exposure to SHS and significant health improvements among employees who work in businesses who comply with smoke-free policies. For instance, after six months’ implementation, 92% of surveyed Scottish bar staff worked in environments without SHS exposure [8]. Following the country’s implementation of smoke-free legislation in Ireland, ambient air nicotine concentrations decreased by 83%, and bar employees’ exposure to SHS plunged from 30 hours per week to zero [9].

Efforts to reduce smoking in public places have a 20-year history in Shanghai, The first "Shanghai Provisional Regulation of No-smoking in Public Places" in Shanghai came in to force in 1994. These initial regulations were amended in 1997 [10]. According to this policy, eight kinds of public places were targeted to become smoke-free but even among these settings smoking was not completely prohibited. For instance, smoking was prohibited in the commercial area of shops larger than 200 square meters. In hospitals, smoking was prohibited only in waiting rooms, diagnosis and treatment rooms, and sickrooms. In school and kindergartens, smoking was prohibited in children’s education and activity areas. Hotels and restaurants were not mentioned in these regulations. In addition to the piecemeal nature of these policies, enforcement of the regulations was not strong. Since the World Health Organization Framework Convention on Tobacco Control [11] officially took effect in China on January 9, 2006, several large Chinese cities including Beijing, Shenyang, Yinchuan, Hangzhou, Guangzhou, and Shanghai have extended their local smoking bans.

In March 2010, the Shanghai Public Places Smoking Control Legislation was passed by the Shanghai People’s Congress Standing Committee and went into effect. This was the first provincial-level legislation on tobacco control passed by the Standing Committee in China. According to this legislation, smoking is totally prohibited in 13 types of public places including medical institutions; inside and outside nurseries and kindergartens; primary and secondary schools; indoor public places for science, education, culture and art; and public transportation areas such as buses and the subway. Since the implementation of these regulations, the Shanghai government has built a management working system that is consistent across seven enforcement authorities [12]. However, the new legislation is still not a comprehensive ban. For instance, no detailed standards were provided for hotel lobbies and restaurants, and smoking zones were still permitted in these settings. In addition, there is still no mention in the policy of workplaces such as offices.

We aimed to evaluate the compliance, support, and impact of new smoke-free legislation in Shanghai after six months of implementation to inform further improvement of this policy. In addition, although evidence from research in other countries showed clear reductions in SHS exposure and improved health among employees [7,13–16], these potential benefits await confirmation in mainland China where some of the aforementioned concerns may dilute findings of decreased exposure. The importance of determining the real impact of this legislation goes beyond Shanghai’s borders. The National Government in China is planning the introduction of country-wide legislation on tobacco control [17]. Further, the experiences and lessons from Shanghai can also work as a reference for other developing countries with a high smoking prevalence.

## Materials and Methods

### Ethics Statement

Ethics approval was granted by the Ethics Committee of the School of Public Health, Fudan University. Written informed consent was obtained from the participants.

### Participants and procedures

Five municipal districts were randomly selected among the 19 districts in Shanghai (Changning, Huangbu, Jingan, Xuhui, Minhang). In each district, two hospitals, two schools (one primary school and one junior high school), and two kindergartens were purposively selected to present areas where people frequently visit or were commonly occupied by local residents. Hotels and shopping malls were randomly selected from the three downtown districts (Changning, Huangbu, and Jingan). In all, there were nine hospitals, nine schools (four primary schools and five junior high schools), 10 kindergartens, 20 hotels, and 11 shopping malls were included in this study. The eligibility criteria for employees included: (1) being 18 years or older, (2) being employed for a minimum of 24 hours per week, (3) being employed in the current workplace for at least 30 days, and (4) working indoors at least five hours per day.

The study was designed as a repeated cross-sectional survey. In each of the selected establishments, we asked the manager to provide a list of staff meeting the above criteria. All employees on the list were approached by our research team to participate in the survey through face-to-face interviews using questionnaires. The first survey was performed in August 2009 [18], about six months prior to the implementation of the new legislation (T1); the follow-up survey was conducted in September 2010, when the legislation had been in place for approximately six months (T2).

In T1, 2,254 participants from 59 work settings participated in the survey with a response rate of 89.9%. In T2,52 of the original 59 settings agreed to participate; 1832 employees completed the questionnaire with an 85.1% response rate.

Managers of two hotels and one shopping mall declined participation in the second questionnaire. The managers told us exposure of SHS was not a serious problem in their work settings and believed there was no need to conduct the survey again. Also, two schools and one kindergarten refused the second survey, with headmasters reporting that staff were too busy in the new semester and had no time to participate. There was also one kindergarten that had been combined with other school so follow-up was not possible. To test the possibility of sample bias due to attrition, comparisons were made on the personal demographic characteristics between participants who only completed the baseline survey and those who completed both surveys. No statistically significant differences were found in demographic variables. Therefore, these data were excluded from the analysis. Altogether, 1907 questionnaires in the baseline and 1832 questionnaires in the follow up were included in the analysis.

### Measures

Assessments included in this study focused on compliance with the policy and the possible impact of knowledge and attitudes, SHS exposure, and short-term health benefits.

Participating employees provided information on demographic characteristics, exposure to SHS, personal smoking behavior, respiratory symptoms, and attitudes towards the public smoking ban. Based on questions about smoking behavior, we categorized employees into two groups: current smokers and nonsmokers. Current smokers were defined as those who reported smoking 100 cigarettes or more in their life and had smoked in the past 30 days.

SHS exposure was assessed by asking, ”In a typical working day, how many hours are you exposed to other people’s tobacco smoke indoors at work?” Respondents who self-reported 0 hours of exposure were classified as not exposed, while the others were classified as exposed and were also operationalized as continuous variables.

Knowledge about the harms of SHS was assessed by asking, “How did exposure to SHS affect the nonsmokers’ health? (1) severely affected; (2) moderately affected; (3) somewhat affected; or (4) did not affect”. We also asked the participants to select diseases related to SHS from multiple choices including lung cancer, cardiovascular disease, bronchitis, and abortion.

Knowledge about the smoking control legislation was assessed by asking, “Do you know about the new smoking control legislation in Shanghai(1)? I heard about it and know at least some specific content; (2) I heard about it but do not know the specific content; or (3) I never heard about it”. Another question is “According to the new smoking control legislation, do you know which of the following is consistent with the regulation in your workplace(1)? smoking is prohibited in all indoor and outdoor areas; (2) smoking is prohibited in all indoor areas; (3) smoking is only allowed in designated smoking zones indoors; or (4) smoking is permitted anywhere”. Responses consistent with the smoking control regulations for their workplace were considered to be correct.

Attitudes toward the smoke-free policy were evaluated by asking, “Do you support smoke-free policy in all workplaces?” with a 5-point scale from (1) strongly agree/supportive to (5) strongly disagree/not supportive.

To measure the compliance with the policy, different questions were developed for smokers and nonsmokers. For smokers, the question was designed as “Which of the following description can best describe your smoking behavior in your indoor workplace? (1) I smoke everywhere; (2) I only smoke in designated smoking zone; or (3) I do not smoke at workplace”.

Nonsmokers were asked, “Would you like to stop other people smoking if you see some visitors smoke in your workplace? (1) absolutely yes; (2) maybe; or (3) absolutely no”. If the answer was absolutely not, they were asked about their reasons for stopping others with multiple choice: (1) smoking by others has nothing to do with me; (2) I have no rights to stop other people smoking; (3) smokers would not follow my advice; (4) avoid disputing with others; or (5) other reasons (specify). Participants were also asked about their behavior if their colleagues smoke in the workplace and about their reasons for not stopping colleagues to smoke. The fourth option was replaced with (4) avoid damage the relationship among colleagues.

Overall compliance with the new legislation was assessed by asking all participants, “According to the new smoking control legislation which has been enforced since March this year, smoking is prohibited indoors in your workplace (or smoking is only allowed in designated smoking zone depending on the workplace regulation). How do you assess the implementation of current regulation (legislation) in your workplace? (1) legislation is enforced most of time; (2) legislation is enforced occasionally; (3) legislation is somewhat enforced; or (4) legislation is not enforced at all”.

The questions on respiratory symptoms were derived from the International Union against Tuberculosis and Lung Disease Bronchial Symptoms Questionnaire [19]. This questionnaire, which has been successfully used in similar studies [20,21], includes five upper respiratory symptoms (wheezing, dyspnea, morning cough, cough during the rest of the day or night, as well as phlegm production) and three sensory symptoms (red or irritated eyes, runny, sneezing nose, and sore or scratchy throat). Respondents were asked if they had experienced any of the above symptoms in the previous 4 weeks.

### Statistical analysis

Data were analyzed with SPSS version 16.0. Fisher’s exact tests and χ^2^ tests were used to examine group differences for categorical variables, and Student t-tests were used to examine differences between groups for continuous data. All subjects were included in estimating workplace exposure to SHS. However, only nonsmokers were included when assessing the proportion of suffering from respiratory symptoms.

## Results

### Demographic information of participants

Table 1 shows the characteristics of the participants. Due to the type of workplaces selected in this study, there was a greater proportion of females than males. No significant differences were found in occupation, gender, or education among the participants across the surveys (P>0.05). However, in T2, the proportion of staff in middle age was higher and the smoking prevalence among participants was lower.

**Table 1:** Workplace and demographic characteristics of participants (%).

	T1(n=1907)	T2 (n=1832)	χ^2^ (P)
**Workplace**			
Hotels	645 (33.8)	629 (34.3)	
Shopping malls	386 (20.2)	377 (20.6)	
Hospitals	445 (23.3)	419 (22.9)	1.95 (0.49)
Schools	290 (15.0)	261 (14.2)	
Kindergartens	141 (7.4)	146 (8.0)	
**Gender**			
Male	656 (34.4)	584 (32.9)	0.90 (0.34)
Female	1251 (65.6)	1190 (67)	
**Age group**			
16-29	837 (43.9)	619 (33.8)	
30-49	885 (46.4)	973 (53.1)	42.44 (<0.001)
50-65	185 (9.7)	240 (13.1)	
**Education**			
Less than middle school	166 (6.1)	126 (7.1)	
High school	622 (32.6)	647 (36.7)	3.59 (0.46)
College and above	1169 (61.3)	982 (55.9)	
**Smoking Prevalence**	453 (23.8)	326 (17.8)	20.12 (<0.01)

### Knowledge of the policy and awareness of harms of SHS

In all, 51.7% of the participants answered that they knew at least some specific content of the new legislation, while 42.7% had heard about it but did not know the details. Participants from the schools and hospital were more likely to be knowledgable about the content of the legislation (65.7% and 60.9%, respectively), whereas employees in hotels and shopping malls were less knowledgable (45.4% and 44.8%).

Fortunately, knowledge about smoking control regulation in their respective workplaces was much better. The proportion of selecting correct option of the smoking control regulation was ranked as following: hospitals (90.8%), kindergartens (87.7%), schools (83.9%), hotels (69.8%), and shopping malls (47.1%). Interestingly, 40.5% of employees in shopping malls selected the option “smoking is only allowed in designated smoking zones”, which is consistent the previous regulation in 1994.

No significant difference were documented in selection of responses to “Exposure to SHS would affect health (including severely or moderately)” before and after the legislation (79.4% vs. 78.4%).

### Support for the policy

Overall, there was sharp increase in support for the total smoking ban among employees in all public places. There were 87.3% of the participants who supported a total ban in their workplaces in T2. The greatest change occurred in hotels, but no obvious change was found in hospitals (Table 2).

**Table 2:** Support for a total ban and evaluation of compliance to current regulation (%).

		T1(n=1907)	T2 (n=1832)	% change (95%CI)
**Support for a total smoking ban in all workplaces**	Hotels	59.1 (55.7,62.9)	81.2 (78.2,84.2)	22.1 (17.0,27.2)**
	Shopping malls	79.0 (76.1,85.1)	86.1 (82.6,89.7)	7.1 (2.0,12.2)**
	Hospitals	89.5 (86.6,92.4)	90.8 (87.9,93.6)	1.3 (-3.3,5.3)
	Schools	85.2 (81.2,89.3)	93.4 (89.8,96.3)	8.2 (3.1,13.3)**
	Kindergartens	88.7 (83.1,93.6)	96.5 (93.2,99.3)	7.8 (2.7,12.9)**
	Total	75.8 (73.9,77.8)	87.3 (6.0,89.0)	11.5 (6.4,16.6)**
**Current regulation (legislation) can be enforced most of time**	Hotels	-	72.2 (66.3,78.1)	
	Shopping malls	57.5 (51.6, 63.4)	69.0 (65.1,72.9)	11.5 (4.6,18.4)*
	Hospitals	59.6 (55.7,63.5)	78.7 (74.8,82.6)	19.1 (13.2,25.0)**
	Schools	72.3 (66.4,78.2)	91.5 (85.6,97.4)	19.2 (13.3, 25.1)**
	Kindergartens	94.8 (90.9,98.7)	92.3 (90.3,94.3)	-2.5 (-6.4,2.4)
	Total **_+_**	65.3 (63.3,67.3)	81.9 (79.9,83.9)	16.6 (13.7,19.5)**

*P<0.05 ;**P<0.01

+ There was no regulation on tobacco use in hotels in T1. To compare the general implementation status, hotels were excluded in the total comparison

### Compliance with the policy

This study indicated that in general, the implementation of the smoke-free policy was better in T2 than that in T1. In T2, 81.9% participants agreed that “current regulation (legislation) can be implemented in most times” (Table 2). However, in hotels and shopping malls, this proportion was about 70%.

Only 29.4% of smokers reported that they did not smoke in their workplaces. This proportion was higher in kindergartens, schools, and hospitals (100%, 63%, and 50%, respectively) but lower in hotels and shopping malls (21.7% and 13.5%, respectively). In all, 65.8% reported they smoked in designated smoking zones, and 4.8% of smokers reported that they smoked anywhere in the workplace. However, according to the new smoking legislation, smoking zones were not allowed in kindergartens, schools, and shopping malls. Given this, 43.2% of smokers did not comply with the smoking control regulation in their workplace.

Table 3 shows prevalence of nonsmokers who would stop other people smoking when seeing someone smoking in the workplace. In all, less than half of nonsmokers would stop visitors from smoking, and the proportion of stopping colleagues was even lower. The proportion of intention to persuade smoking visitors was the highest in hospitals and lowest in shopping malls (70% and 38.9%, respectively). The reasons for not stopping visitors from smoking ranked as follows: avoiding disputing with others (49.7%), having no rights to persuade others (38.8%), smokers would not follow my advice (31.4%), smoking by others has nothing to do with me (12.5%), other reasons (3.2%). The reasons for not stopping colleagues from smoking were similar; “Avoid damaging the relationship among colleagues” was the most frequently cited reason (44.8%).

**Table 3:** The proportion of non-smoking participants selecting “I would stop other people smoking in the workplace” (%).

	If seeing visitors smoking	If seeing colleagues smoking
Hotels	45.3 (40.6,49.9)	42.4 (37.7,47.2)
Shopping malls	39.0 (32.6,45.0)	15.8 (11.3,20.6)
Hospitals	71.0 (66.2,75.5)	55.1 (49.6,60.4)
Schools	40.2 (33.8,46.7)	40.2 (33.9,46.3)
Kindergartens	51.5 (43.3,60.3)	55.1 (46.9,63.6)
Total	50.2 (47.4,53.0)	41.8 (39.2,44.3)

### Exposure to SHS

The overall prevalence of exposure to SHS decreased from 48.9% to 35.6% while the exposure time decreased from 1.16±1.97h/d to 0.94±1.69h/d. The largest reduction to SHS occurred in hospitals and schools, whereas kindergartens kept low exposure levels in both surveys. Exposure to SHS in hotels and shopping malls remained high at T2 (Table 4).

**Table 4:** Daily exposure to secondhand smoke in the workplace before and after legislation.

	Exposure rate (%)		Exposure time (x±s,h/d)	
	T1	T2	T1	T2
Hotels	49.0 (45.1,52.9)	39.2 (35.2,43.2)*	1.23±2.01	1.19±1.86*
Shopping malls	64.0 (60.1,67.9)	53.0 (47.1,58.9)*	1.79±2.47	1.24±1.73**
Hospitals	46.9 (43.0,50.8)	27.0 (23.1,30.9)**	1.11±1.71	0.62±1.40**
Schools	21.8 (19.8,23.8)	8.5 (4.6,12.4)**	0.30±0.62	0.13±0.55*
Kindergartens	0.5 (-1.5,2.5)	0.7 (-1.3,2.7)	0.01±0.07	0.01±0.10
Total	48.9 (46.9,50.9)	35.6 (31.7,39.5)**	1.16±1.97	0.94±1.69*

* P<0.05 ; **P<0.01

### Health benefits

Overall, there was a significant decrease in prevalence of all respiratory symptoms. There were statistically significant differences by type of establishment: shopping malls and schools had a sharp decrease in respiratory and sensory symptoms (P<0.05). However, there was no significant change in kindergartens after the legislation (Table 5).

**Table 5:** Prevalence (%) of respiratory and sensory symptoms among employees before and after legislation.

	Wheeze		Shortness of breath		Morning cough		Frequent cough		Phlegm		Sore eyes		Runny nose		Sore throat		Any symptoms	
	T1	T2	T1	T2	T1	T2	T1	T2	T1	T2	T1	T2	T1	T2	T1	T2	T1	T2
Hotels	6.9	3.2**	18.6	9.4**	11.7	8.3*	19	9.4**	31.4	19.6**	24.8	16.4**	32.3	19.1**	46.4	27.7**	81.1	71.4**
Shopping Malls	8.0	2.1**	21.1	5.6**	18.8	10.6**	19.4	8.1**	38.5	22.3**	25.2	9.8**	33.1	19.1**	51.5	30**	85.4	69.8**
Hospitals	1.0	0.9	5.2	4.1	7.3	8.1	8.8	7.9	18.7	16.3	16.0	8.4**	25.5	21	37.1	25.5**	59.8	52.2*
Schools	0.6	0.8	5.4	1.5*	6.4	7.3	9.2	6.1	22.6	14.9*	7.3	4.2*	15.0	12.6	28.0	23.4*	64.9	53.6**
Kindergartens	1.4	0.7	5.6	2.1	10.4	8.9	4.2	8.9	22.2	15.8**	11.1	6.2	17.4	27.4	30.6	32.2	68.3	68.0
Total	4.3	1.9**	12.5	5.7**	11.1	8.6*	14	8.2**	27.6	18.8**	18.1	10.6**	25.6	19.3**	39.4	27.4**	82.9	67.0**

* P<0.05 and **P<0.01

## Discussion

To our knowledge, this is the first comprehensive evaluation of a provincial-level smoking control policy in mainland China. A reduction in exposure to SHS was seen half a year after the regulation was enacted. This study also confirms increasing support for a total smoking ban in the workplaces among employees. This study clearly demonstrates that the introduction of legislation in Shanghai has led to a decrease in exposure to SHS in these workplaces. These findings are consistent with those in other countries [22–25].

This study is somewhat different from other studies examining the impact of smoke-free legislation [14,26,27]. In fact, there were some pre-existing regulations in schools, kindergartens, shopping malls, and hospitals [10]. Therefore, this study is not a “none to all” comparison. In addition, the results presented in this study suggest that the implementation of this policy was not as effective as in other studies. Compared to other international studies [13–16,28,29], the results of this study showed lower compliance rates, higher proportions of exposure to SHS, and limited health effects.

The content of the policy is crucial in setting parameters for implementation [30]. Effectiveness of smoke-free laws is greatly weakened when smoking is permitted in designated areas [31–33]. One of the important reasons that the Shanghai government did not introduce a comprehensive smoke-free policy is that the policy-makers regarded the implementation of a “Smoking control” policy as more practical and feasible in Shanghai than a total ban [34]. However, this study showed that in kindergartens and schools, the workplaces with the strictest regulations on tobacco use, the implementation of the policy was better than other public places, with more than 90% of staff in these workplaces agreeing that the legislation could be implemented most or all of the time. On the other hand, in hotels which were legislated to have only a partial smoking ban, implementation was not optimal and only 72.2% of the staff in this type of workplace agreed that the legislation could be enforced in their workplace most of time. In the current legislation, it is stated that separate rooms (smoking zones) can be established in the public areas of hotels without providing any further details or standards. The results of this study challenge the popular misunderstanding in China that a partial ban is more feasible in practice.

This study also indicated limited understanding of the current smoking legislation. About half participants did not know the details of the legislation. About half of participants in hotels and shopping malls had inaccurate knowledge of regulations for their workplace. This may be due to two reasons. First, the legislation is complicated with details about where smoking is allowed. Second, the media communication activities surrounding the legislation are insufficient. Experience from other countries shows that education and advocacy efforts increased public support for 100% smoke-free policies and decreased the social acceptability of smoking [35].

The successful implementation of smoking control legislation required compliance from smokers and supporting behaviors from nonsmokers. This study showed that 43.2% of smokers could not comply with the smoking regulation. Therefore, increasing the compliance among smokers is the first step with a possible solution being increasing the amount of the fine which is only 50 RMB (about $8) according to the current legislation. In addition, targeting the social norms of smoking and decreasing social acceptability of smoking by health advocacy is an important approach for promoting compliance with smoking restrictions in China where smoking is widely accepted [36,37]. Last but not least, the managers in workplaces should take the corresponding opportunity in educating staff and enforcing the rule. Roughly two-thirds of smokers still smoked in the designated indoor smoking zones. This reveals that designated smoke zones still existed in some workplaces despite being not allowed according to the new legislation.

On the other hand, less than half of nonsmokers would stop visitors from smoking in their workplace. The main reason included avoiding disputes with others, feeling as though they have no rights to persuade others, and concerns that smokers would not follow the advice. This partly reflects the culture of China to keep harmony and avoid disputes [38]. To change the status, training should be provided to staff on how to stop others smoking which can efficiently avoid disputes and increase the self-efficacy among the nonsmokers. The conception such as “breathing fresh air is the right if everyone” should be advocated among nonsmokers. In addition, as business places, both the hotels and shopping malls give high priority to the customers’ perceived rights to smoke. Results here indicate that managers in these workplaces felt that limiting smoking among customers may have a negative impact on business. However, many international studies have shown that hospitality sectors do not experience any negative economic effects and may even experience some positive effects after the introduction of smoke-free laws [31,39]. Such evidence in China is still necessary to dispel such doubts.

Although compliance is a challenge, this study found increasing support for a total ban for smoking after the legislation. The support rate for a total smoking ban in all workplaces increased from 75.8% to 87.3%. This is consistent with other studies which also showed increased social support and acceptability to smoke-free policies after the legislation was enacted [25,33,40]. Our study found that employees in kindergartens, schools, and hospitals had higher support rates than other groups. However, the greatest change to support rates to a total ban occurred in hotels, with no significant change occurring among working staff in hospitals. Hotels had virtually no regulations on smoking before the legislation. Therefore, employees in hotels may have experienced the greatest change over the course of the study. However, according to the current legislation, smoking was only partially banned in hotels so there is room to further push toward a smoke-free environment. Hospitals, on the other hand, had pre-existing smoking regulations before the new legislation, with a high support rate among the staff. Because working staff in hospitals have more knowledge on the harms of SHS, those who did not support the smoke-free policy in hospitals may be harder to change than other working populations.

There are several limitations in our study. First, we chose to use an anonymous questionnaire design to encourage truthful reporting among the employees. However, such methods also excluded the possibility to assess the change in individuals over time. Second, recall bias in the perception of exposure to SHS as well as in the reporting of respiratory symptoms cannot be disregarded. Although other studies have shown that self-reports are consistent with conclusions from environmental monitoring and biomarker studies [32,41], objective measurements of exposure to SHS such as cotinine level or concentration of particulate matter should be applied in the future. Third, seven worksites dropped out from T1 to T2 in this study. Worksite self-selection bias may have affected the results of this study. Last but not least, this study only assessed short-term impact; a longer term follow up survey should be conducted in the future.

## Conclusion

Results of this study confirm a reduction in exposure to secondhand smoke and decreased prevalence of suffering from respiratory and sensory symptoms after the implementation of smoking control legislation in Shanghai. This study also indicates increased public support for such policies. Further efforts should be engaged in to strengthen the enforcement of current legislation and to decrease the social acceptability of smoking.
